# D-4F, an apolipoprotein A-I mimetic, promotes the clearance of myelin debris and the reduction of foamy macrophages after spinal cord injury

**DOI:** 10.1080/21655979.2022.2073063

**Published:** 2022-05-12

**Authors:** Jinxin Li, Zhenyu Zhu, Yiteng Li, Yihao Chen, Xuyang Hu, Yanchang Liu, Yi Shi, Yao Hu, Yihui Bi, Xinzhong Xu, Meige Zheng, Li Cheng, Juehua Jing

**Affiliations:** Department of Orthopedics, The Second Affiliated Hospital of Anhui Medical University, Hefei, Anhui, China

**Keywords:** D-4F, spinal cord injury, foamy macrophages, myelin debris, ABCA1

## Abstract

After spinal cord injury (SCI), a large number of blood-derived macrophages infiltrate the lesion site and phagocytose myelin debris to become foamy macrophages, which leads to chronic inflammation. The drug D-4F, an apolipoprotein A-I peptidomimetic made of D-amino acids, has been reported to promote the lipid metabolism of foamy macrophages in atherosclerosis. However, the role and mechanism of D-4F in SCI are still unclear. In this study, we found that D-4F can promote the removal of myelin debris, reduce the formation of foamy macrophages in the lesion core and promote neuroprotection and recovery of motor function after SCI. These beneficial functions of D-4F may be related to its ability to upregulate the expression of ATP-binding cassette transporter A1 (ABCA1), the main transporter that mediates lipid efflux in foamy macrophages because inhibiting the activity of ABCA1 can reverse the effect of D-4F *in vitro*. In conclusion, D-4F may be a promising candidate for treating SCI by promoting the clearance of myelin debris by foamy macrophages via the ABCA1 pathway.

## Graphical abstract

**Figure:**
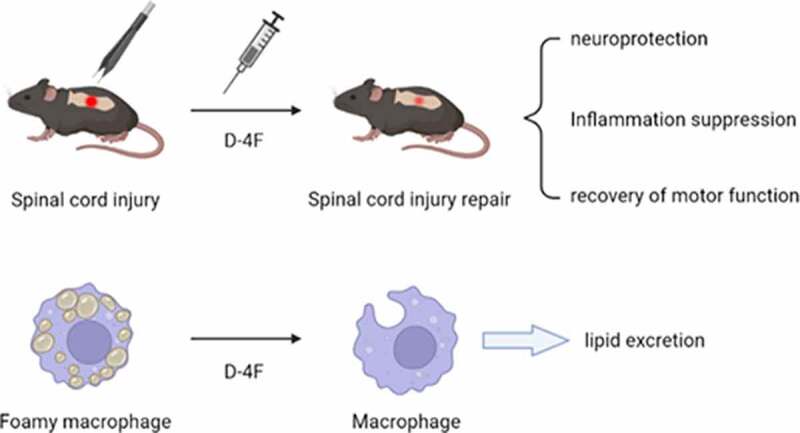

## Highlights

- D-4F promotes the clearance of myelin debris after spinal cord injury.
- D-4F promotes the reduction of foamy macrophages after spinal cord injury.
- D-4F improves the neurological and motor functions after spinal cord injury.
- D-4F upregulated the expression of ABCA1 after spinal cord injury.
- D-4F may function via the ABCA1 pathway after spinal cord injury.

## 1. Introduction

Spinal cord injury (SCI) is a serious central nervous system (CNS) injury characterized by cell death and demyelination [1], resulting in the accumulation of myelin debris at the injury site, accompanied by the activation of resident microglia and infiltration of blood-derived macrophages [2–4]. Recent studies have shown that infiltrating blood-derived macrophages can effectively phagocytose myelin debris [5]. However, their lipid efflux function, mainly mediated by ATP-binding cassette transporter A1 (ABCA1) export to apolipoproteins, appears to be disturbed, leading to lipid accumulation in the cytoplasm and the formation of foamy macrophages fully packed with lipid droplets in the lesion core (LC) [6]. Foamy macrophages gradually lose the ability to migrate and produce proinflammatory cytokines, leading to necrosis and chronic inflammation [7,8]. Therefore, reducing the accumulation of myelin debris and the formation of foamy macrophages, thereby inhibiting the continuation of inflammation, helps to restore function after SCI.

D-4F, an apolipoprotein A-I (APOA-I) peptidomimetic made of D-amino acids, can promote cholesterol efflux in macrophages via the cAMP-PKA-ABCA1 pathway [9]. It can also promote polarization of M2-type macrophages to treat stroke in rats with type one diabetes [10] and pass through the blood–brain barrier for the treatment of Alzheimer’s disease [11]. Furthermore, it has been proven to play a role in the treatment of neurogenic inflammation [12], inflammation of cerebral arterioles [13], and other diseases.

We hypothesized that D-4F may contribute to the clearance of myelin debris in the treatment of SCI. In this study, we established a mouse model of SCI, and the mice were treated by intraperitoneal injection with D-4F to investigate its function on foamy macrophages and the specific mechanism. We found that D-4F can inhibit the formation of foamy macrophages and promote the clearance of myelin debris in the LC. In addition, it can promote neuroprotection and recovery of motor function. Mechanistically, the ABCA1 pathway was found to play an indispensable role in the treatment of D-4F. These findings provide a new therapeutic direction for the treatment of SCI.

## 2. Materials and methods

### 2.1. Animals and cells

All animal experiments complied with the ARRIVE guidelines and were performed in accordance with the the National Institutes of Health Guide for the Care and Use of Laboratory Animals and were approved by the Institutional Animal Care and Use Committee of Anhui Medical University (approval No. LLSC20160052). Specific pathogen-free female C57BL/6 J mice (weighing 20–25 g and aged 8–12 weeks) were obtained from the Animal Experiment Center of Anhui Medical University (license No. SYXK (Wan) 2017–006) and were raised in the Research Center of the Second Affiliated Hospital of Anhui Medical University. The temperature and humidity of the breeding environment were strictly controlled, and a 24 h day-night cycle with a 1:1 light: dark ratio was maintained. The mice could freely obtain sufficient water and food. Cultured mouse mononuclear macrophage leukemia RAW 264.7 cells in Dulbecco’s modified Eagle’s medium (HyClone, Invitrogen, United States) containing 10% fetal bovine serum (Gibco, United States), 1% GlutaMAX and 1% sodium pyruvate (Invitrogen, United States) were provided by the Stem Cell Bank of the Chinese Academy of Sciences (Shanghai, China). The culture environment of RAW 264.7 cells was 5% CO2 and 37°C.

### 2.2. Establishment of the mouse SCI model

The mice were anesthetized with 50 mg/kg pentobarbital sodium (P-010; Sigma, St. Louis, Missouri, United States) and underwent surgery in the prone position. The back hair was shaved, and the skin was disinfected with iodophor 3 times. All surgical instruments were autoclaved. A longitudinal incision in the skin was made at the level of T10 and extended down until the lamina was exposed. A rongeur was used to remove part of the lamina of T10 until the spinal cord was exposed. Fine forceps with a width of 0.5 mm at the tip (511,252–20, Fine Science Tools, Heidelberg, Germany) were used to apply pressure from both sides of the spinal cord for 5 seconds [14]. During the clamping process, the spinal cord was clamped completely and the same clamping pressure was maintained (with the tips of the fine forceps just touching as the standard), resulting in moderate compression injury. To reduce the influence of interfering factors on the functional recovery of mice, those with poor postoperative states or severe complications were excluded as experimental subjects. After the operation, the bladder was pressed to assist the mice in urinating twice a day. On the first day after surgery, D-4F was continuously injected intraperitoneally (dissolved in phosphate-buffered saline (PBS) at a dose of 1 mg/kg/day per day), while the control groups were injected with the same amount of PBS.

### 2.3. Basso Mouse Scale (BMS) behavioral analysis

BMS was used to assess the recovery of motor function in mice after SCI [15]. Each mouse was placed in the open field one day before the operation to become familiar with the environment, and the BMS score was determined from 1 day postinjury (dpi) to 28 dpi. The observation period of the score was 4 minutes. All mice were observed and assessed by three independent researchers blinded to the groups, and the final result was averaged. The BMS scale ranges from 0 (no ankle joint movement) to 9 (equivalent to normal animals) and is based on hind limb joint movement, trunk stability, gait coordination, paw and tail position and stability.

### 2.4. Preparation of myelin debris and DiO-myelin

Myelin debris was extracted from the brains of 8- to 12-week-old mice, and the obtained myelin debris solution was purified by sucrose gradient centrifugation without using detergent. The specific steps were to purify with 0.32 M and 0.83 M sterile sucrose solution and Tris. Cl buffer, respectively. Then, the solution was centrifuged at 4°C for 45 min at 100,000 × g and 10 min at 22,000 × g in an ultracentrifuge and adjusted to a final concentration of 100 mg/ml [8]. According to the needs of different experiments, the working concentration of myelin debris was diluted to 0.125–1.0 mg/ml [16]. 3,3’-Dioctadecyloxacarbocyanine perchlorate (DiO, C1993S, Beyotime Biotechnology, China) was added to label the prepared myelin debris solution, which was mixed thoroughly and incubated for 20 min at 37°C in the dark. The final DiO-myelin preparation was successful and was mixed with cells.

### 2.5. Immunofluorescence staining

The mice were anesthetized by intraperitoneal injection of 50 mg/kg pentobarbital sodium and placed in the supine position, and the thoracic cavity was opened to expose the heart and perfused with PBS and 4% paraformaldehyde (4% PFA, Servicebio, China) to remove the blood from the spinal cord. Forceps were used to carefully remove the target spinal cord segment (including the injured core, approximately 5 mm), which was completely immersed and fixed in 4% PFA for 6 h. Then, the segment was soaked in 30% sucrose solution for 24 h to dehydrate the tissue. Subsequently, the tissue was embedded with OCT embedding agent (Sakura, United States), and the embedded tissue was sliced into 16-micron-thick serial sections at −30°C on a cryostat (NX50, Thermo Fisher, United States) and attached to glass slides. Sagittal sections containing the lesion core were used. The sections were dried at 50°C for 2 h, washed 3 times with PBS, blocked with blocking solution containing 5% donkey serum (SL050, Solarbio, China) and 0.3% Triton X-100 in PBS at room temperature for 1 h, and incubated with the following primary antibodies overnight at 4°C: rabbit anti-ABCA1 (1:100, NB400-105, Novus Biologicals, United States), goat anti-5-hydroxytryptamine (5-HT) (1:5000, 20,079, Immunostar, United States), rat anti-glial fibrillary acidic protein (GFAP) (1:200, 133–0300, Invitrogen, United States), rat anti-Mac2 (1:100, sc-23,938, Santa Cruz, United States), rabbit anti-degraded myelin basic protein (dMBP) (1:100, AB5864, Chemicon, United States), or rabbit anti-neurofilament heavy polypeptide (NFH) (1:500, ab7206, Abcam, United Kingdom). The sections were then washed 3 times with PBS and incubated with the following secondary antibodies for 1 h at 37°C in the dark: donkey anti-rabbit Alexa Fluor 594, donkey anti-rabbit Alexa Fluor 555, donkey anti-rat Alexa Fluor 594, donkey anti-goat Alexa Fluor 594, and donkey anti-rat Alexa Fluor 488 (1:500, A-21207, A-11058, A-21209, A-21208, Invitrogen, United States). The sections were then washed 3 times with PBS and dried. Finally, an anti-fluorescence quencher (P0126, Beyotime Biotechnology Company, China) was used to cover the tissues, and 4ʹ6-diamidino-2-phenylindole staining was performed. A Zeiss LSM 900 confocal microscope system and fluorescence microscope (AxioScopeA1, Zeiss, Germany) were used to ensure that the light intensity of all images was the same, and representative images were obtained through comparison and analysis. ImageJ (v1.53c, National Institutes of Health, Bethesda, MD, USA) was used to perform the quantitative analysis.

### 2.6. Immunocytochemistry staining

The treated cells were fixed with 4% PFA for 15 min, washed 3 times with PBS, and blocked with 5% donkey serum albumin for 30 min at room temperature. The primary antibody was incubated overnight at 4°C with rat anti-Mac2. The cells were washed 3 times with PBS and incubated with the secondary antibody in the dark. Finally, the cover glass was sealed with an anti-fluorescence quencher. Representative images were obtained through comparison and analysis by fluorescence microscopy.

### 2.7. Oil Red O (ORO) staining

ORO staining was performed to detect the accumulation of myelin debris in the LC after SCI and the accumulation of myelin debris in RAW 264.7 cells. For cell experiments, RAW 264.7 cells were treated with myelin debris or D-4F, and the concentration gradient settings of the two were based on previous studies [17]. The cells to be treated were first washed twice with PBS and fixed with an ORO fixative (G1262, Solarbio, China) for 20–30 min. The fixative was discarded, and the cells were washed twice with distilled water and stained with ORO staining solution (G1260, Solarbio, China) for 20 min. The staining solution was discarded, and the cells were washed 3 times with distilled water. Hematoxylin was added to counterstain the nucleus for 2 min. Finally, ORO buffer was added for 1 min, and the cover glass was sealed with distilled water. For tissue experiments, frozen sections of the spinal cord were dried and washed with PBS, stained with ORO staining solution for 10 min, treated with 60% isopropanol for 5 min, and finally washed with distilled water. Representative images were obtained by fluorescence microscopy.

### 2.8. Western blot assay

To obtain the cell protein, the cells in a 6-well plate were washed 3 times with PBS, and protease inhibitor cocktail and phosphatase inhibitor (04693124001, 04906845001, Roche, Switzerland) in radioimmunoprecipitation assay buffer (P0013B, Beyotime Biotechnology, China) were mixed sufficiently and added to the 6-well plate with shaking on ice for 30 min. For the spinal cord tissue protein, the target spinal cord segment (3 mm) including the injury core was obtained after cardiac perfusion with PBS, and the aforementioned lysis buffer was added to lyse the cells, which were also smashed by abrasive drilling. After centrifugation at 4°C and 13,000 rpm, the supernatant was collected for protein quantification using a bicinchoninic acid kit (B0012S, Beyotime Biotechnology Company, China). LDS sample buffer (NP0007, Invitrogen, United States) was added to the sample protein and heated at 100°C for 10 min to achieve thermal denaturation. Then, the protein sample was resolved by gel electrophoresis separation and transferred onto polyvinylidene difluoride membranes. The membranes were blocked in 5% skim milk for 1 h, and then the target membranes were incubated with the following primary antibodies overnight: rabbit anti-ABCA1, rabbit anti-myelin basic protein (MBP) (1:1000, BA0094, Boster, China) and rabbit anti-β-tubulin (1:3000, DF7967, Affinity, United States). The membranes were washed three times with PBS and incubated with the following secondary antibody for 1 h at room temperature: goat anti-rabbit (1:10,000, A0545, Sigma, United States). A chemiluminescence detection kit (Thermo Fisher Scientific) and Tanon 5200 system (Tanon, China) were used to obtain protein band signals. The quantitative analysis results were obtained via ImageJ (NIH, United States) and are shown.

### 2.9. Imaging analysis and quantification

To evaluate the accumulation of myelin debris in foamy macrophages, they were divided into 3 levels, +, ++ and +++, representing the proportions of myelin debris occupying < 1/3, 1/3–2/3 and > 2/3 of the cytoplasm, respectively. The sum of the proportion of cells occupied by ++ and +++ was used for statistical analysis. Using Zeiss ZEN imaging software (Zeiss) and a 40× objective, the number of cells in each grade was counted (n ≥ 75 cells in each group), and the percentage of myelin-laden foamy macrophages was calculated [17]. To quantify the accumulation of lipids in the LC after SCI, the proportion of the dMBP^+^ area to the area within 300 μm from the LC was calculated, and the position of the box was kept unchanged. The proportion of the ORO^+^ area to the observed field of view was calculated. To quantify tissue repair and the regeneration of neurofilaments and axons, the dashed line enclosed the GFAP^−^ area and the ratio of the GFAP^−^ area to the sum of the GFAP^−^ area and the GFAP^+^ area was calculated. Box selection was within a 300 μm range from the LC, and the position of the box was unchanged. Then, the ratio of the NFH^+^ area in the selected area to the area of the selected area was calculated. Taking the rostral and caudal ends of the tissue within 500 μm from the LC as the selected field of view, the proportion of 5-HT^+^ area in the selected field of view was calculated, and nonspecific staining at the edge of the tissue was excluded. At least 3 animals were tested in each of the above groups, and at least 5 sections of the entire spinal cord of each animal were uniformly and intermittently selected for staining [18].

### 2.10. Statistical analysis

Each group of experiments was repeated independently at least 3 times, with n indicating the number of experiments, and the blinded samples were quantified. The data are expressed as the mean ± standard error of the mean (SEM). SPSS version 19.0 (IBM SPSS Inc., Chicago, IL, United States) was used to perform the statistical analysis. Comparisons between two groups were performed using Student’s t test. Analysis of variance (ANOVA) followed by Tukey’s *post hoc* test was used to compare multiple-group differences. *P* < 0.05 indicated that the difference was statistically significant.

## 3. Results

In this study, immunofluorescence staining, ORO staining, Western blot assays and other techniques were conducted to investigate the role of D-4F in SCI. The results showed that D-4F can promote the removal of myelin debris in the center of injury, the reduction of foamy macrophages, and the recovery of motor function. Moreover, the expression of ABCA1 was upregulated, which may be the main mechanism by which D-4F works.

### 3.1. * D-4F promotes the excretion of myelin debris from foamy macrophages* in vitro

**Figure 1.**
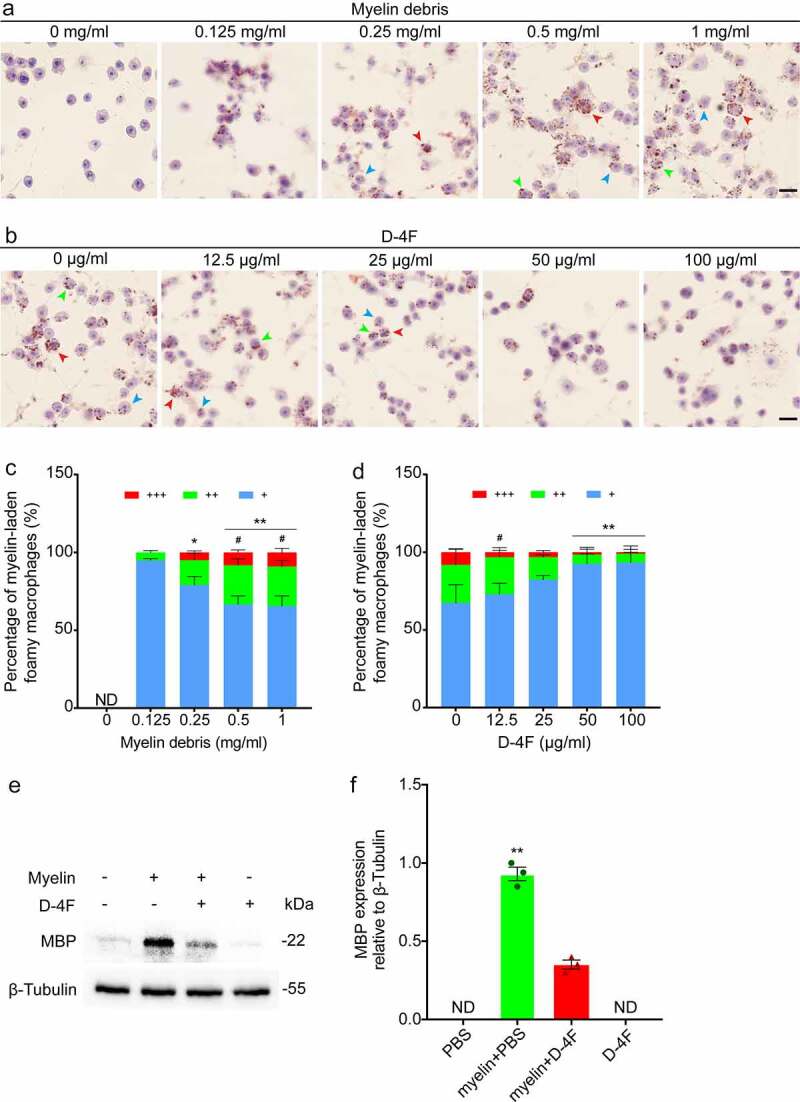
RAW 264.7 cells treated with myelin debris and D-4F *in vitro*. (a) RAW 264.7 cells were incubated with different concentrations of myelin debris for 24 h and stained with ORO, and the cell nuclei were stained with hematoxylin. (b) RAW 264.7 cells were incubated with 0.5 mg/ml myelin debris for 24 h, treated with different concentrations of D-4F for 24 h and stained with ORO. The blue, green and red arrowheads indicate typical myelin-laden foamy macrophages, representing myelin debris occupying < 1/3, 1/3–2/3 and > 2/3 of the cytoplasm, respectively. Scale bars, 20 μm (a-b). (c-d) Quantitative analysis of the percentage of myelin-laden foamy macrophages formed at different concentrations of myelin debris and D-4F. The extent of myelin-laden foamy macrophage formation was divided into 3 levels, +, ++ and +++, representing the proportions of myelin debris occupying < 1/3, 1/3–2/3 and > 2/3 of the cytoplasm, respectively. The proportion of foamy macrophages with myelin debris occupying more than 1/3 of the cytoplasm was compared between the groups; that is, the sum of the proportion of cells occupied by ++, +++ was used for statistical analysis. The results are shown as the means ± SEM. (c) *p < 0.05, **p < 0.01 (0.25, 0.5 and 1 mg/ml vs. 0.125 mg/ml); *^#^p* < 0.05 (0.5 and 1 mg/ml vs. 0.25 mg/ml), one-way ANOVA followed by Tukey’s *post hoc* test. ND, not detected. (d) **p < 0.01 (50 and 100 μg/ml vs. 0 μg/ml); *^#^p* < 0.05 (12.5 μg/ml vs. 50 and 100 μg/ml), one-way ANOVA followed by Tukey’s *post hoc* test. (e) Western blotting was used to detect the expression level of MBP in RAW 264.7 cells after treatment with 0.5 mg/ml myelin debris and 50 μg/ml D-4F. (f) Quantitative analysis of ABCA1 expression in (E). The blots (n = 3 per group) were quantified by a densitometric method using ImageJ software. The corresponding quantification of protein levels was determined by densitometry analysis relative to β-tubulin. The results are shown as the means ± SEM. **p < 0.01 (myelin + PBS vs. myelin + D-4F, one-way ANOVA followed by Tukey’s *post hoc* test). ND, not detected.

To test the effect of D-4F on the removal of myelin debris from macrophages, we first treated RAW 264.7 cells with different concentration gradients of 0–1 mg/ml myelin debris and constructed a foamy macrophage model *in vitro*. (Figure 1(a)). ORO staining showed that as the concentration of myelin debris increased, more myelin debris was phagocytosed by RAW 264.7 cells. When the concentration reached 0.5 mg/ml (*p* < 0.05, vs. 0.25 mg/ml; Figure 1(c)), which was close to saturation, the proportion of foamy macrophages with myelin debris in the cytoplasm occupying more than 1/3 of the cytoplasm no longer increased with increasing concentrations of myelin debris. Next, we set up different concentration gradients of 0–100 μg/ml D-4F (Figure 1(b)) at a myelin debris concentration of 0.5 mg/ml to explore the effect of D-4F on foamy macrophages. The quantitative analysis results showed that as the concentration of D-4F in the medium increased, the proportion of foamy macrophages with myelin debris accounting for more than 1/3 of the cytoplasm gradually decreased until the concentration of D-4F reached 50 μg/ml (*p* < 0.01, vs. 0 μg/ml; Figure 1(d)), and the proportion no longer changed significantly. The Western blotting results indicated that the content of myelin debris marked by MBP was downregulated after treatment of foamy macrophages with D-4F (*p* < 0.01, vs. Myelin + D-4F; Figure 1(e,f)).

**Figure 2.**
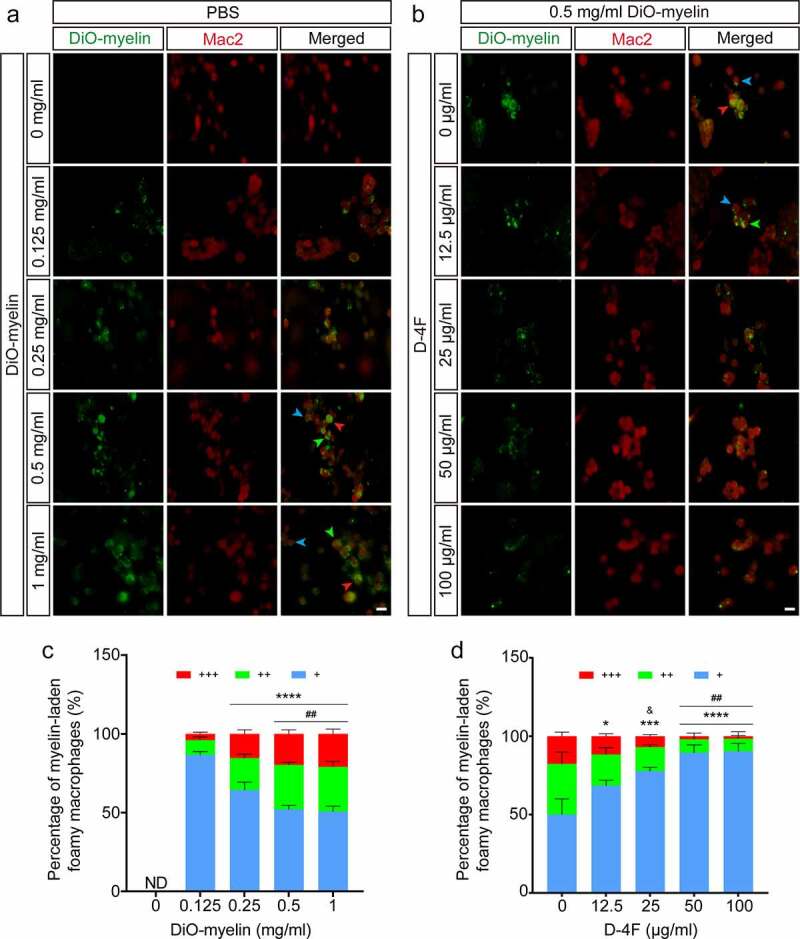
RAW 264.7 cells treated with DiO-myelin and D-4F *in vitro*. (a) RAW 264.7 cells were incubated with different concentrations of DiO-myelin for 24 h. Immunocytochemistry staining was used to label macrophages (Mac2^+^, red). (b) RAW 264.7 cells were incubated with 0.5 mg/ml DiO-myelin for 24 h and then treated with different concentrations of D-4F for 24 h. Immunocytochemistry staining was used to label macrophages (Mac2^+^, red). The blue, green and red arrowheads indicate typical myelin-laden foamy macrophages, representing myelin debris occupying < 1/3, 1/3–2/3 and > 2/3 of the cytoplasm, respectively. Scale bars, 20 μm (a-b). (c-d) Quantitative analysis of the percentage of myelin-laden foamy macrophages formed at different concentrations of DiO-myelin and D-4F. Similarly, the extent of myelin-laden foamy macrophage formation was divided into 3 levels: +, ++ and +++, DiO-myelin occupying < 1/3, 1/3–2/3 and > 2/3 of the cytoplasm, respectively. The results are shown as means ± SEM. The proportion of foamy macrophages with DiO-myelin occupying more than 1/3 of the cytoplasm was compared between the groups; that is, the sum of the proportion of cells occupied by ++, +++ was used for statistical analysis. (c) ****p < 0.0001 (0.25, 0.5 and 1 mg/ml vs. 0.125 mg/ml); *^##^p* < 0.01 (0.5 and 1 mg/ml vs. 0.25 mg/ml), one-way ANOVA followed by Tukey’s *post hoc* test. ND, not detected. (d) *p < 0.05, ***p < 0.001, ****p < 0.0001 (12.5, 25, 50 and 100 μg/ml vs. 0 μg/ml); *^##^p* < 0.01 (50 and 100 μg/ml vs. 12.5 μg/ml); *^&^p* < 0.05 (25 μg/ml vs. 50 and 100 μg/ml), one-way ANOVA followed by Tukey’s *post hoc* test.

Next, we used immunocytochemistry staining for further verification. Similarly, the quantitative analysis results showed that as the concentration of DiO-myelin increased, more Mac2^+^ cells phagocytized myelin debris (Figure 2(a)). When the concentration of myelin debris reached 0.25 mg/ml (*p* < 0.0001, vs. 0.125 mg/ml; Figure 2(c)), the proportion of foamy macrophages with myelin debris accounting for more than 1/3 of the cytoplasm increased significantly. It reached saturation when the concentration reached 0.5 mg/ml (*p* < 0.01, vs. 0.25 mg/ml; Figure 2(c)). Then, we set the concentration of DiO-myelin to 0.5 mg/ml and treated 5 groups with different concentrations of D-4F (Figure 2(b)). From the quantitative analysis results, when the concentration of D-4 F was between 12.5 μg/ml and 50 μg/ml (*p* < 0.05, *p* < 0.001, *p* < 0.0001 vs. 0 mg/ml, respectively; Figure 2(d)), the higher the concentration of D-4F was, the more myelin debris was discharged by foamy macrophages. When the concentration of D-4F exceeded 50 μg/ml, the effects did not change significantly. Thus, RAW 264.7 cells phagocytosing myelin debris may reach saturation at a myelin debris concentration of 0.5 mg/ml at 24 h, while D-4F can promote the excretion of myelin debris from foamy macrophages, and 50 μg/ml may be the optimal concentration of D-4F *in vitro*.

### 3.2. D-4F promotes the clearance of myelin debris and the reduction of foamy macrophages in the LC after SCI in vivo

**Figure 3.**
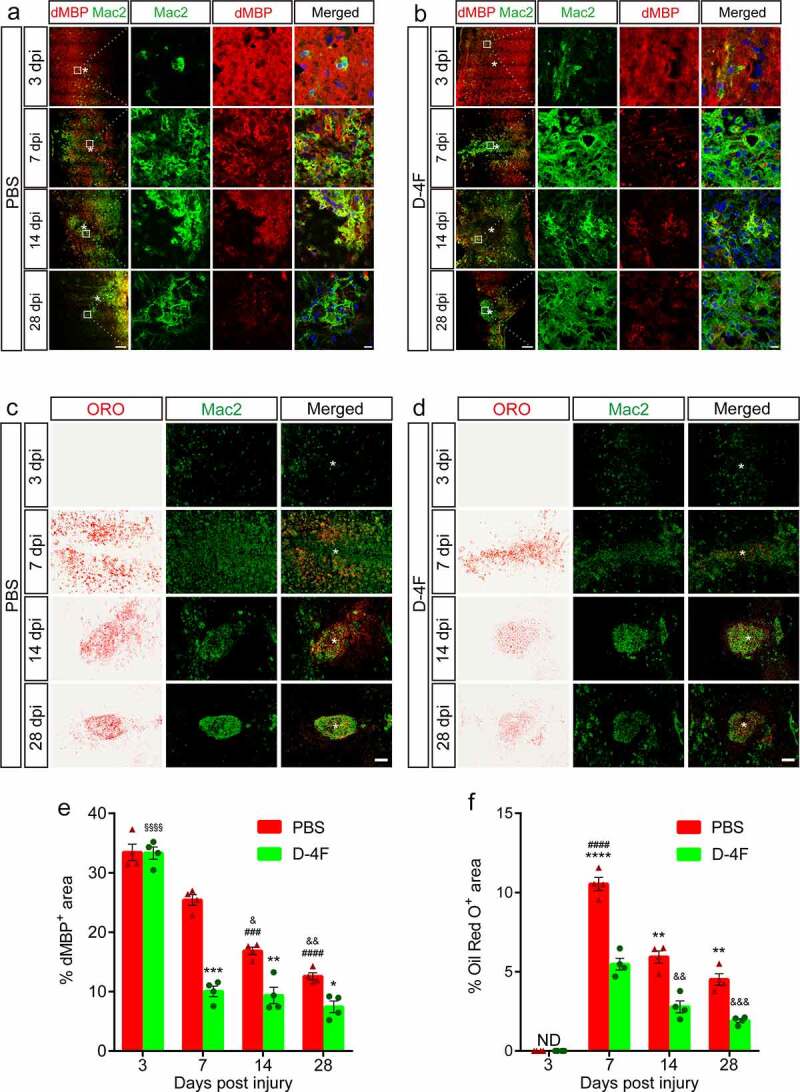
D-4F can promote the removal of myelin debris from the LC after SCI. (a-b) Representative images of immunofluorescence staining of dMBP (red) and Mac2 (green) in the LC at the indicated time points after SCI. The mice were treated by intraperitoneal injection with PBS in (a) and with D-4F in (b). The asterisks indicate the lesion epicenter. Scale bars, 200 μm (low-magnification images), 10 μm (high-magnification images). (c-d) Representative images of double staining for Mac2 (green) and ORO (red) in the LC at the indicated time points after SCI. The mice were treated by intraperitoneal injection with PBS in (c) and with D-4F in (d). The asterisks indicate the lesion epicenter. Scale bars, 100 μm (C-D). (e) Quantitative analysis of the dMBP^+^ area in the PBS and D-4F groups at each time point after SCI (n = 4 per group). ***p < 0.001, **p < 0.01, *p < 0.05 (D-4F vs. PBS at 7,14 and 28 dpi, respectively); *^###^p* < 0.001, *^####^p* < 0.0001 (14 and 28 dpi vs. 3 dpi in PBS, respectively); *^&^p* < 0.05, *^&&^p* < 0.01 (14 and 28 dpi vs. 7 dpi in PBS, respectively); *^§§§§^p* < 0.0001 (3 dpi vs. 7, 14 and 28 dpi in D-4F), two-way ANOVA followed by Tukey’s *post hoc* test. (f) Quantitative analysis of the ORO^+^ area in the PBS and D-4F groups at each time point after SCI (n = 4 per group). The results are shown as the means ± SEM. ****p < 0.0001, **p < 0.01 (PBS vs. D-4F at 7, 14 and 28 dpi); *^####^p* < 0.0001 (7 dpi vs. 14 and 28 dpi in PBS); *^&&^p* < 0.01, *^&&&^p* < 0.001 (14 and 28 dpi vs. 7 dpi in D-4F, respectively), two-way ANOVA followed by Tukey’s *post hoc* test. ND, not detected.

To investigate the effect of D-4F on the accumulation of myelin debris in the LC after SCI *in vivo*, a rabbit anti-dMBP antibody was used to specifically label degraded myelin basic protein [19], and a rat anti-Mac2 antibody was used to label macrophages [7]. The immunofluorescence staining results showed a slow decreasing trend in dMBP from 3 dpi to 28 dpi in the LC in the control group (Figure 3(a)), and the D-4F group also showed a decreasing trend (Figure 3(b)). Although no significant difference was found between the data of the two groups at 3 dpi, the dMBP levels in the D-4F group decreased sharply at 7 dpi and were much lower than those of the control group (*p* < 0.001, vs. D-4F; Figure 3(e)) after 7 dpi. We used immunofluorescence staining combined with ORO staining [7] to observe the positioning relationship between macrophages and myelin debris accumulation in the LC. The results showed that myelin debris stained by ORO staining and Mac2 labeled by immunofluorescence staining were roughly colocalized (Figure 3(c,d)), which indicated that most of the myelin debris in the LC was phagocytosed by macrophages. From 7 dpi to 28 dpi, the ORO^+^ area gradually decreased in both groups, but it was significantly smaller in the D-4F group than in the control group at each time point (*p* < 0.0001 and *p* < 0.01, vs. D-4F, respectively; Figure 3(f)). These results suggested that D-4F can promote the clearance of myelin debris and the reduction of foamy macrophages in the LC after SCI *in vivo*.

### 3.3. D-4F improves the neurological and motor functions of mice after SCI

**Figure 4.**
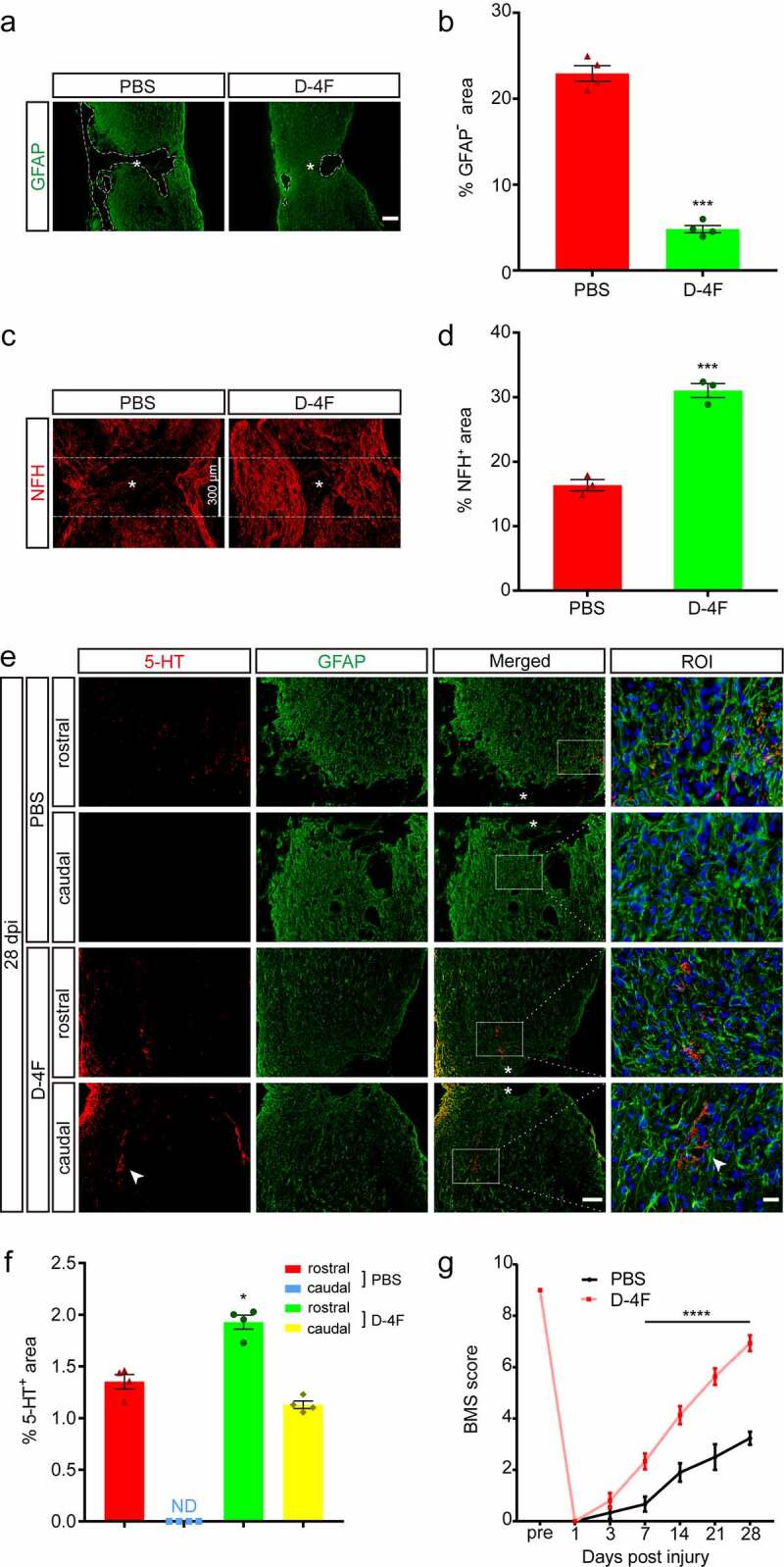
D-4F can promote tissue repair, neuroprotection and motor function recovery in SCI model mice. (a) Representative images of immunofluorescence staining of GFAP (green) at 4 weeks after SCI. The mice were treated by intraperitoneal injection with PBS and D-4F. The parts enclosed by the dotted lines represent the area of GFAP^−^ in the LC. The asterisks indicate the lesion epicenter. Scale bar, 200 μm. (b) Quantitative analysis of the GFAP^−^ area in the PBS and D-4F groups at 4 weeks after SCI (n = 4 per group). ***p < 0.001 (D-4F vs. PBS, Student’s t test). (c) Representative images of immunofluorescence staining of NFH (red) in the LC at 4 weeks after SCI. The mice were treated by intraperitoneal injection with PBS and D-4F. The asterisks indicate the lesion epicenter, and 150 μm areas on both sides of the lesion epicenter are separated by dotted lines. Scale bar, 300 μm. (d) Quantitative analysis of the proportion of the NFH^+^ area in the area enclosed by the dotted line in the PBS and D-4F groups at 4 weeks after SCI (n = 3 per group). ***p < 0.001 (D-4F vs. PBS, Student’s t test). (e) Representative images of immunofluorescence staining of 5-HT (red) and GFAP (green) at 4 weeks after SCI. The mice were treated by intraperitoneal injection with PBS and D-4F. Approximately 500 μm zones abutting the LC were selected as the rostral and caudal ends. The region of interest (ROI) represents the boxed region on the left and shows detailed immunostaining of 5-HT and GFAP. The arrowheads indicate the typical area of 5-HT^+^. The asterisks indicate the lesion epicenter. Scale bars, 100 μm (low magnification images), 20 μm (high magnification images). (f) Quantitative analysis of the 5-HT^+^ area in the PBS and D-4F groups at the rostral and caudal ends at 4 weeks after SCI (n = 4 per group). *p < 0.05 (D-4F vs. PBS at the rostral end, two-way ANOVA followed by Tukey’s *post hoc* test). ND, not detected. (g) Statistical analysis of the BMS score in the PBS and D-4F groups over a 28-day period (n = 5 mice per group at each time point). The results are shown as the means ± SEM. ****p < 0.0001 (D-4F vs. PBS at 7, 14, 21 and 28 dpi, two-way ANOVA followed by Tukey’s *post hoc* test).

We further detected the effects of D-4F treatment on organizational retention, neuroregeneration and functional recovery in an SCI model. The immunofluorescence staining results showed that the percentage of the GFAP-negative area in the D-4F group was much smaller than that in the control group at 28 dpi (*p* < 0.001, vs. D-4F; Figure 4(a,b)), indicating that D-4F can reduce the area of damage and promote tissue repair. The NFH-positive area in the D-4F group was much greater than that in the control group (*p* < 0.001, vs. PBS; Figure 4(c,d)), indicating that D-4F can promote the regeneration of NF axons in the LC. The 5-HT axons in the control group gathered at the rostral end of the spinal cord [20] and failed to penetrate the core of the injury at 28 dpi (Figure 4(e)). The distribution of 5-HT was clearly seen at the caudal end of the spinal cord in the D-4F group, and the expression of 5-HT at the rostral end was also greater than that in the control group (*p* < 0.05, Figure 4(f)), indicating that D-4F can help 5-HT axons regenerate and pass through the LC. The BMS score was recorded and used to evaluate the two groups of mice for 28 consecutive days, and we found no significant difference in the scores of the two groups before 3 dpi. The difference between the two groups gradually became obvious, and the BMS score of the D-4F group was much higher than that of the control group after 7 dpi (*p* < 0.0001, vs. PBS, respectively; Figure 4(g)). The highest score of the control group before 28 dpi was approximately 3 points, while that of the D-4F group reached close to 7 points. The motor function of SCI mice was also improved under D-4F treatment. In summary, D-4F plays an important role in neuroprotection and recovery of motor function after SCI.

### 3.4. - D-4F may promote the expression of ABCA1 in macrophages in the LC

**Figure 5.**
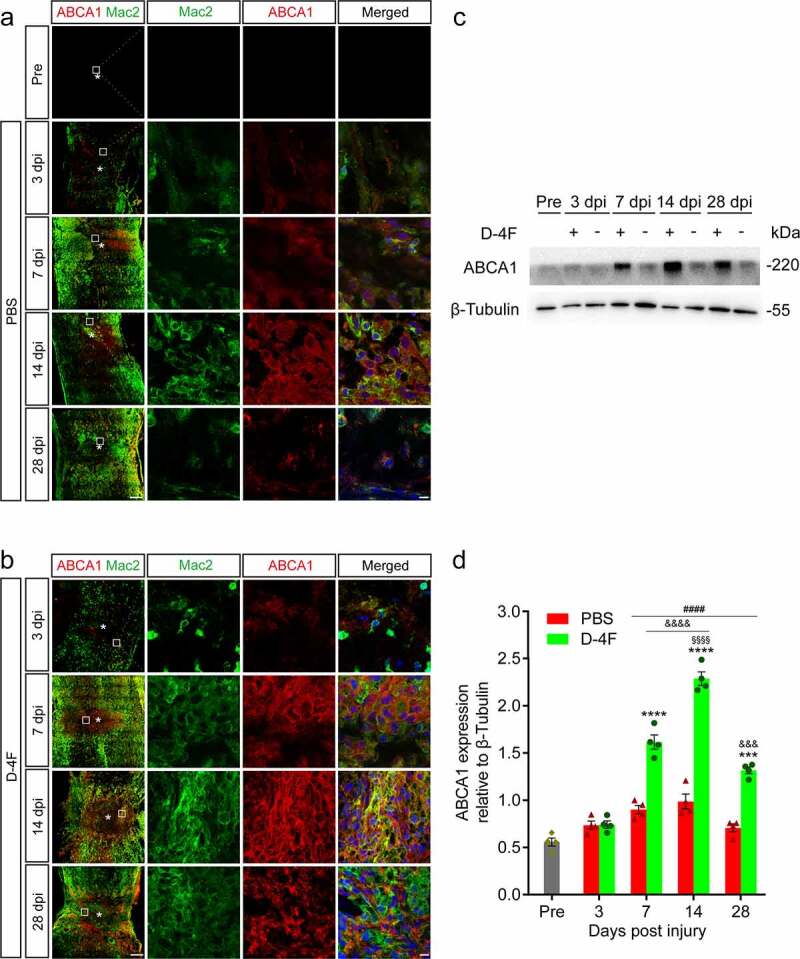
D-4F promotes the expression of ABCA1 in macrophages in the LC. (a-b) Representative images of immunofluorescence staining of ABCA1 (red) and Mac2 (green) in the LC at the indicated time points after SCI. The mice were treated by intraperitoneal injection with PBS in (a) and with D-4F in (b). The region of interest (ROI) represents the boxed region on the left and shows detailed immunostaining of ABCA1 and Mac2. The asterisks indicate the lesion epicenter. Scale bars, 200 μm (low-magnification images), 10 μm (high-magnification images). (c) Western blotting was used to detect the expression levels of ABCA1 at the indicated time points after SCI in mice. (d) Quantitative analysis of ABCA1 expression in (C). The blots (n = 4 per group) were quantified by a densitometric method using ImageJ software. The corresponding quantification of protein levels was determined by densitometry analysis relative to β-tubulin. The results are shown as the means ± SEM. ****p < 0.0001, ***p < 0.001 (D-4F vs. PBS at 7, 14 and 28 dpi, respectively); *^####^p* < 0.0001 (7, 14 and 28 dpi vs. Pre in D-4F, respectively); *^&&&&^p* < 0.0001, *^&&&^p* < 0.001 (7, 14 and 28 dpi vs. 3 dpi in D-4F); *^§§§§^p* < 0.0001 (14 dpi vs. 7 and 28 dpi in D-4F), two-way ANOVA followed by Tukey’s *post hoc* test.

APOA-I plays an important role in promoting the clearance of myelin debris through the ABCA1 pathway in macrophages [21]. To further investigate the mechanism of D-4F in SCI, immunofluorescence staining and Western blotting were used to detect the expression and positioning of ABCA1 in the injured spinal cord from a preoperative time point to 28 dpi. From the immunofluorescence staining results, whether in the control group or the D-4F group, ABCA1 colocalized with Mac2 (Figure 5(a,b)), and the distribution of ABCA1-positive areas in the D-4F group appeared to be greater than that in the control group. The Western blotting results suggested that the expression of ABCA1 gradually increased from 3 dpi to 14 dpi after SCI and decreased after 14 dpi. The expression of ABCA1 in the D-4F group rose sharply at 7 dpi and was much greater than that of the control group, peaked at 14 dpi, and still maintained its high expression level at 28 dpi (*p* < 0.0001, *p* < 0.001, vs. PBS, respectively; Figures 5(c,d)). Although the overall trend in the control group was roughly the same as that of the D-4F group, it was much lower. In general, the above results indicated that D-4F upregulated the expression of ABCA1 and may function via the ABCA1 pathway in the lipid efflux of foamy macrophages in the LC after SCI.

### 3.5. D-4F may promote the clearance of myelin debris through the ABCA1 pathway

**Figure 6.**
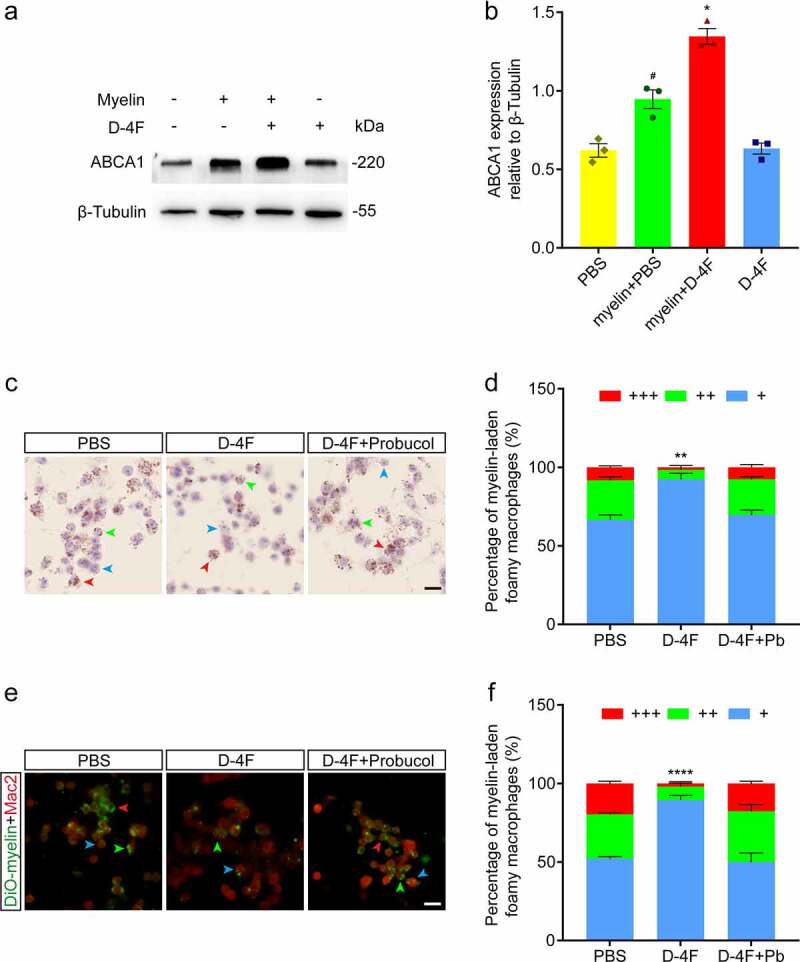
D-4F may promote the clearance of myelin debris through the ABCA1 pathway. (a) Western blotting was used to detect the expression level of ABCA1 in RAW 264.7 cells after treatment with 0.5 mg/ml myelin debris and 50 μg/ml D-4F. (b) Quantitative analysis of ABCA1 expression in (A). The blots (n = 3 per group) were quantified by a densitometric method using ImageJ software. The corresponding quantification of protein levels was determined by densitometry analysis relative to β-tubulin. The results are shown as the means ± SEM. *^#^p* < 0.05 (myelin + PBS vs. PBS); *p < 0.05 (myelin + D-4F vs. myelin + PBS, one-way ANOVA followed by Tukey’s *post hoc* test). (c) RAW 264.7 cells were incubated with 0.5 mg/ml myelin debris for 24 h, treated with D-4F (50 μg/ml) and probucol (Pb, 10 μM), and then stained with ORO. The cell nuclei were stained with hematoxylin. The blue, green and red arrowheads indicate typical myelin-laden foamy macrophages, with myelin debris occupying < 1/3, 1/3–2/3 and > 2/3 of the cytoplasm, respectively. Scale bars, 20 μm. (d) Quantitative analysis of the percentage of myelin-laden foamy macrophages formed under different conditions. The extent of myelin-laden foamy macrophage formation was divided into 3 levels: +, ++ and +++, representing myelin debris occupying < 1/3, 1/3–2/3 and > 2/3 of the cytoplasm, respectively. The proportion of foamy macrophages with myelin debris occupying more than 1/3 of the cytoplasm was compared between the groups; that is, the sum of the proportion of cells occupied by ++, +++ was used for statistical analysis. The results are shown as the means ± SEM. **p < 0.01 (D-4F vs. PBS and D-4F + Pb, one-way ANOVA followed by Tukey’s *post hoc* test). (e) RAW 264.7 cells were incubated with 0.5 mg/ml DiO-myelin for 24 h and treated with D-4F (50 μg/ml) and probucol (10 μM). Immunocytochemistry staining was used to label macrophages (Mac2^+^, red). The blue, green and red arrowheads indicate typical myelin-laden foamy macrophages, with myelin debris occupying < 1/3, 1/3–2/3 and > 2/3 of the cytoplasm, respectively. Scale bars, 20 μm. (f) Quantitative analysis of the percentage of myelin-laden foamy macrophages formed under different conditions in (E). The same method was used as in (D). The results are shown as the means ± SEM. ****p < 0.0001 (D-4F vs. PBS and D-4F + Pb, one-way ANOVA followed by Tukey’s *post hoc* test).

We further verified whether D-4F promoted the clearance of myelin debris by foam macrophages through the ABCA1 pathway *in vitro*. The Western blotting results indicated that after RAW 264.7 cells were incubated with myelin debris for 24 h, the expression of ABCA1 was upregulated (*p* < 0.05, vs. PBS; Figures 6(a,b)). Another group was incubated with myelin debris for 24 h and then treated with D-4F for 24 h, and the expression of ABCA1 exceeded that of the previous group (*p* < 0.05, vs. Myelin + PBS; Figures 6(a,b)). We established a control group with only D-4F to exclude the influence of D-4F on RAW 264.7 cells that had not phagocytized myelin debris. These results suggested that D-4F can upregulate the expression of ABCA1 in foamy macrophages. Then, the ABCA1 inhibitor probucol was used to reduce the activity of ABCA1 such that the channel could not function [22]. ORO staining (*p* < 0.01, D-4F vs. PBS and D-4F + Pb; Figures 6(c,d)) and immunocytochemistry (*p* < 0.0001, D-4F vs. PBS and D-4F + Pb; Figures 6(e,f)) were used to detect lipid efflux in foamy macrophages treated with D-4F and probucol. After the activity of ABCA1 was reduced, D-4F lost its ability to promote lipid efflux by foamy macrophages. Based on the above results, we propose that D-4F may promote the clearance of myelin debris by foamy macrophages via the ABCA1 pathway.

## 4. Discussion

After SCI, the accumulation of myelin debris leads to the formation and aggregation of foamy macrophages. In this study, we found that the APOA-I peptidomimetic D-4F can promote the upregulation of ABCA1 by macrophages, thereby accelerating the clearance of myelin debris, reducing the formation of foamy macrophages, and ultimately promoting neuroprotection and recovery of motor function.

To date, no quantitative analysis of the accumulation of myelin debris in the LC on different specific days after SCI has been performed. The accumulation of myelin debris in the LC of an impact model of SCI at 14 dpi has been reported to be greater than that at 7 dpi [7]. This indicates that the myelin debris produced by demyelination continuously increased before 14 dpi, and the speed of increase was greater than the speed of removal. The above results have not been quantitatively analyzed. In this study, we found that from 3 dpi to 28 dpi, the accumulation of myelin debris in the LC showed a downward trend, and the decline in the D-4F treatment group was much greater than that in the control group. These results indicated that the removal rate of myelin debris in the LC was greater than the rate of demyelination after 3 dpi. This is different from the results of previous studies. The reason may be the different degrees of SCI caused by the clamp model and the impact model, which lead to a difference in the amount of myelin debris accumulated in the LC. The accumulation of myelin debris in the LC may be related to the mechanism or the degree of SCI, and our results reveal that D-4F treatment is beneficial in promoting the clearance of myelin debris.

Although recent studies have shown that microvascular endothelial cells can be used as ‘amateur’ phagocytic cells to engulf myelin debris [16], ‘professional’ phagocytes, including infiltrating macrophages and resident microglia, still play a major role. The inflammatory reaction caused by physical stimulation and spinal cord hemorrhage will quickly activate resident microglia to perform phagocytosis before 3 dpi, and then recruited blood-derived macrophages will take over this role and perform phagocytosis predominantly after 3 dpi. Although microglia are more efficient at processing CNS debris than macrophages, phagocytosed material cannot be detected in microglia after 3 dpi, while that in macrophages can last up to 42 dpi [5]. Therefore, we used foamy macrophages as our research object to find a breakthrough that can promote the removal of myelin debris in the LC. Consistent with previous studies [7], our study confirmed that macrophages accumulate in large numbers at 7 dpi and continue gradually accumulating toward the center of the injury until 28 dpi (Figure 3(a)).

The excretion channels of cholesterol in macrophages include ABCA1, ABCG1 and other secondary channels [23]. Among them, ABCA1 is the main pathway of lipid excretion: two lipid-free APOA-I molecules bind to ABCA1 dimers such that the lipids in macrophages are transported out in the form of high-density lipoprotein (HDL) [24]. After macrophages phagocytose myelin debris, due to insufficient expression of ABCA1, lipid efflux is blocked, resulting in the formation of foamy macrophages and lipid plaques. ABCA1 has been reported to have the highest expression level at 7 dpi and be significantly reduced after 14 dpi. In this study, we found that the expression level of ABCA1 gradually increased from 3 dpi, peaked at 14 dpi, and then began to decline. This is somewhat different from the results of previous studies, which is possibly due to differences in models. After 7 dpi, the expression level of ABCA1 in the D-4F group was much higher than that in the control group. In summary, D-4F significantly upregulated the expression of ABCA1 after 7 dpi. Along with the removal of myelin debris, the number of foamy macrophages and the area of the injured region were reduced, and neuroprotection and functional recovery were enhanced.

The known effects of D-4F on macrophages are as follows: in atherosclerosis models, D-4F can improve HDL-mediated cholesterol efflux in macrophages and reverse cholesterol transport in macrophages, thereby reducing lesions, and its molecular pathway is cAMP-PKA-ABCA1 [9,25]. In rat models of type one diabetes mellitus with stroke, D-4F can increase the expression of microRNA-124 and promote anti-inflammatory effects and M2 macrophage polarization in the brain, which contribute to the improvement of nerve function [10]. Compared with previous studies, our study explored the effect of D-4F on macrophages in SCI models for the first time.

Thus far, some studies have focused on inhibiting the phagocytosis of myelin by macrophages, which can improve the treatment of SCI. For example, the scavenger receptor CD36 was knocked out in mice, the lipid droplet content in macrophages was reduced, and the formation of foamy macrophages was reduced, which resulted in reduced lesion area and better functional recovery [6]. In addition, signals such as CD200 [6], CD31 [26], and plasminogen activator inhibitor (PAI)-1 [27] help inhibit the phagocytosis of macrophages, and they may become new targets for the treatment of SCI in the future. Other studies have shown that promoting the elimination of lipids in macrophages is a beneficial direction for treating SCI. For example, Toll-like receptor 4 (TLR4) is considered a key signaling receptor for the phagocytic ability of macrophages after SCI. Enhancing TLR4 signaling can stimulate the clearance of myelin debris by macrophages and increase the number of remyelinated axons [28]. Furthermore, peroxisome proliferator activator receptor (PPAR-γ), an inducer of ABCA1, was activated by allicin to stimulate lipid efflux in macrophages and reduce the formation of foam cells [29]. Similarly, atorvastatin can indirectly upregulate ABCA1 by activating PPAR-γ to promote functional recovery [30]. In this study, that D-4F may directly upregulate the expression of ABCA1, promote lipid excretion in macrophages, and play a role in treating SCI was demonstrated for the first time.

Previous studies have shown that after SCI, depletion of blood-derived macrophages results in decreased Tnfsf8 and Tnfsf13 (tumor necrosis factor superfamily members) and increased BMP1-7 (bone morphogenetic proteins) expression. This leads to antifibrosis, inhibits fibrotic scar formation, and is ultimately conducive to axonal growth [20]; on the other hand, limiting inflammation, especially the activation of microglia/macrophages, can exert neuroprotective effects [31]. In our study, D-4F was found to promote the removal of myelin debris from the LC and inhibit inflammation, activate macrophages to reduce the accumulation of foamy macrophages, and inhibit the formation of fibrotic scars to facilitate neuroprotection. Due to the inhibition of chronic inflammation and preservation of neural tissue, D-4F treatment may protect NF and 5-HT axons from degeneration rather than promote axon regeneration. Whether it can promote axon regeneration requires further verification.

## 5. Conclusion

Based on the above results, our research discovered a drug, D-4F, for the treatment of SCI. D-4F was confirmed to promote the clearance of myelin debris, the reduction in foamy macrophages in the LC, neuroprotection and the recovery of motor function after SCI. These findings provide new research directions and evidence for the treatment of SCI.

## References

[1] Ahuja CS, Nori S, Tetreault L, et al. Traumatic spinal cord injury-repair and regeneration. Neurosurgery. 2017;80(3S):S9–S22. 28350947 10.1093/neuros/nyw080

[2] Ren Y, Young W. Managing inflammation after spinal cord injury through manipulation of macrophage function. Neural Plast. 2013;2013:945034. 24288627 10.1155/2013/945034 PMC3833318

[3] Skaper SD, Facci L, Giusti P. Neuroinflammation, microglia and mast cells in the pathophysiology of neurocognitive disorders: a review. CNS Neurol Disord Drug Targets. 2014;13(10):1654–1666. 25470401 10.2174/1871527313666141130224206

[4] Bradbury EJ, Burnside ER. Moving beyond the glial scar for spinal cord repair. Nat Commun. 2019;10(1):3879. 31462640 10.1038/s41467-019-11707-7 PMC6713740

[5] Greenhalgh AD, David S. Differences in the phagocytic response of microglia and peripheral macrophages after spinal cord injury and its effects on cell death. J Neurosci. 2014;34(18):6316–6322. 24790202 10.1523/JNEUROSCI.4912-13.2014 PMC6608120

[6] Zhu Y, Lyapichev K, Lee DH, et al. Macrophage transcriptional profile identifies lipid catabolic pathways that can be therapeutically targeted after spinal cord injury. J Neurosci. 2017;37(9):2362–2376. 28130359 10.1523/JNEUROSCI.2751-16.2017 PMC5354348

[7] Wang X, Cao K, Sun X, et al. Macrophages in spinal cord injury: phenotypic and functional change from exposure to myelin debris. Glia. 2015;63(4):635–651. 25452166 10.1002/glia.22774 PMC4331228

[8] Sun X, Wang X, Chen T, et al. Myelin activates FAK/Akt/NF-kappaB pathways and provokes CR3-dependent inflammatory response in murine system. PLoS One. 2010;5(2):e9380. 20186338 10.1371/journal.pone.0009380 PMC2826415

[9] Xie Q, Zhao SP, Li F. D-4F, an apolipoprotein A-I mimetic peptide, promotes cholesterol efflux from macrophages via ATP-binding cassette transporter A1. Tohoku J Exp Med. 2010; 220(3):223–228. 20208418 10.1620/tjem.220.223

[10] Ning R, Venkat P, Chopp M, et al. D-4F increases microRNA-124a and reduces neuroinflammation in diabetic stroke rats. Oncotarget. 2017;8(56):95481–95494. 29221142 10.18632/oncotarget.20751 PMC5707036

[11] Handattu SP, Garber DW, Monroe CE, et al. Oral apolipoprotein A-I mimetic peptide improves cognitive function and reduces amyloid burden in a mouse model of Alzheimer’s disease. Neurobiol Dis. 2009;34(3):525–534. 19344763 10.1016/j.nbd.2009.03.007 PMC5558244

[12] Oehler B, Kloka J, Mohammadi M, et al. D-4F, an ApoA-I mimetic peptide ameliorating TRPA1-mediated nocifensive behaviour in a model of neurogenic inflammation. Mol Pain. 2020;16 :1744806920903848. 31996074 10.1177/1744806920903848 PMC6993174

[13] Buga GM, Frank JS, Mottino GA, et al. D-4F decreases brain arteriole inflammation and improves cognitive performance in LDL receptor-null mice on a Western diet. J Lipid Res. 2006;47(10):2148–2160. 16835442 10.1194/jlr.M600214-JLR200

[14] McDonough A, Monterrubio A, Ariza J, et al. Calibrated forceps model of spinal cord compression injury. J Vis Exp. 2015;(98):52318 . 10.3791/52318 PMC4541603 25938880

[15] Basso DM, Fisher LC, Anderson AJ, et al. Basso mouse scale for locomotion detects differences in recovery after spinal cord injury in five common mouse strains. J Neurotrauma. 2006;23(5):635–659. 16689667 10.1089/neu.2006.23.635

[16] Zhou T, Zheng Y, Sun L, et al. Microvascular endothelial cells engulf myelin debris and promote macrophage recruitment and fibrosis after neural injury. Nat Neurosci. 2019;22(3):421–435. 30664769 10.1038/s41593-018-0324-9 PMC6913093

[17] Yang L, Yang JB, Chen J, et al. Enhancement of human ACAT1 gene expression to promote the macrophage-derived foam cell formation by dexamethasone. Cell Res. 2004;14(4):315–323. 15353128 10.1038/sj.cr.7290231

[18] Anderson MA, Burda JE, Ren Y, et al. Astrocyte scar formation aids central nervous system axon regeneration. Nature. 2016;532(7598):195–200. 27027288 10.1038/nature17623 PMC5243141

[19] Ihara M, Polvikoski TM, Hall R, et al. Quantification of myelin loss in frontal lobe white matter in vascular dementia, Alzheimer’s disease, and dementia with Lewy bodies. Acta Neuropathol. 2010;119(5):579–589. 20091409 10.1007/s00401-009-0635-8 PMC2849937

[20] Zhu Y, Soderblom C, Krishnan V, et al. Hematogenous macrophage depletion reduces the fibrotic scar and increases axonal growth after spinal cord injury. Neurobiol Dis. 2015;74:114–125. 25461258 10.1016/j.nbd.2014.10.024 PMC4323620

[21] Paul A, Lydic TA, Hogan R, et al. Cholesterol acceptors regulate the lipidome of macrophage foam cells. Int J Mol Sci. 2019;20(15):3784 . 10.3390/ijms20153784 PMC6695943 31382484

[22] Arakawa R, Tsujita M, Iwamoto N, et al. Pharmacological inhibition of ABCA1 degradation increases HDL biogenesis and exhibits antiatherogenesis. J Lipid Res. 2009;50(11):2299–305. 19458386 10.1194/jlr.M900122-JLR200 PMC2759836

[23] Chistiakov DA, Bobryshev YV, Orekhov AN. Macrophage-mediated cholesterol handling in atherosclerosis. J Cell Mol Med. 2016;20(1):17–28. 26493158 10.1111/jcmm.12689 PMC4717859

[24] Phillips MC. Is ABCA1 a lipid transfer protein? J Lipid Res. 2018;59(5):749–763. 29305383 10.1194/jlr.R082313 PMC5928442

[25] Navab M, Anantharamaiah GM, Reddy ST, et al. Oral D-4F causes formation of pre-beta high-density lipoprotein and improves high-density lipoprotein-mediated cholesterol efflux and reverse cholesterol transport from macrophages in apolipoprotein E-null mice. Circulation. 2004;109(25):3215–3220. 15197147 10.1161/01.CIR.0000134275.90823.87

[26] Hoek RM, Ruuls SR, Murphy CA, et al. Down-regulation of the macrophage lineage through interaction with OX2 (CD200). Science. 2000;290(5497):1768–1771. 11099416 10.1126/science.290.5497.1768

[27] Park YJ, Liu G, Lorne EF, et al. PAI-1 inhibits neutrophil efferocytosis. Proc Natl Acad Sci U S A. 2008;105(33):11784–11789. 18689689 10.1073/pnas.0801394105 PMC2575264

[28] Church JS, Milich LM, Lerch JK, et al. E6020, a synthetic TLR4 agonist, accelerates myelin debris clearance, Schwann cell infiltration, and remyelination in the rat spinal cord. Glia. 2017;65(6):883–899. 28251686 10.1002/glia.23132 PMC5518478

[29] Lin XL, Hu HJ, Liu YB, et al. Allicin induces the upregulation of ABCA1 expression via PPARgamma/LXRalpha signaling in THP-1 macrophage-derived foam cells. Int J Mol Med. 2017;39(6):1452–1460. 28440421 10.3892/ijmm.2017.2949 PMC5428973

[30] Bensinger SJ, Tontonoz P. Integration of metabolism and inflammation by lipid-activated nuclear receptors. Nature. 2008;454(7203):470–477. 18650918 10.1038/nature07202

[31] Orr MB, Gensel JC. Spinal cord injury scarring and inflammation: therapies targeting glial and inflammatory responses. Neurotherapeutics. 2018;15(3):541–553. 29717413 10.1007/s13311-018-0631-6 PMC6095779

